# Analysis of the Proteins Secreted from the *Oryza meyeriana* Suspension-Cultured Cells Induced by *Xanthomonas oryzae* pv. *oryzae*

**DOI:** 10.1371/journal.pone.0154793

**Published:** 2016-05-19

**Authors:** Xian Chen, Yan Dong, Chulang Yu, XianPing Fang, Zhiping Deng, Chengqi Yan, Jianping Chen

**Affiliations:** 1 State Key Laboratory Breeding Base for Zhejiang Sustainable Pest and Disease Control, MOA Key Laboratory of Biotechnology in Plant Protection, Zhejiang Provincial Key Laboratory of Plant Virology, Institute of Virology and Biotechnology, Zhejiang Academy of Agricultural Science, Hangzhou, 310021, China; 2 College of Agriculture and Biotechnology, Zhejiang University, Hangzhou, 310029, China; 3 College of Chemistry and Life Sciences, Zhejiang Normal University, Jinhua, 321004, China; 4 College of Plant Protection, Nanjing Agricultural University, Nanjing, 210095, China; 5 College of Plant Protection, Hunan Agricultural University, Changshao, 410128, China; Shanghai Jiao Tong University, CHINA

## Abstract

*Oryza meyeriana*, a wild species of rice from China, shows high resistance to *Xanthomonas oryzae* pv. *oryzae* (Xoo), the cause of rice bacterial blight, one of the most serious rice pathogens. To better understand the resistance mechanism, a proteomic study was conducted to identify changes in the proteins secreted in embryo cell suspension cultures in response to Xoo. After two-dimensional difference gel electrophoresis (2D-DIGE), 72 differentially expressed protein spots corresponding to 34 proteins were identified by Matrix-Assisted Laser Desorption/ Ionization Time of Flight Mass Spectrometry. Of the 34 proteins, 10 were up regulated and 24 down regulated. The secreted proteins identified were predicted to be involved in various biological processes, including signal transduction, defense, ROS and cell wall modification. 77% of the 34 proteins were predicted to have a signal peptide by Signal P. Quantitative Real-Time PCR showed that transcript levels of 14 secreted proteins were not well correlated with secreted protein levels. Peroxidase activity was up regulated in both *O*. *meyriana* and susceptible rice but was about three times higher in *O*. *meyeriana*. This suggests that peroxidases may play an important role in the early response to Xoo in *O*. *meyeriana*. These results not only provide a better understanding of the resistance mechanism of *O*. *meyeriana*, but have implications for studies of the interactions between other plants and their pathogens.

## 1 Introduction

Bacterial blight (BB) of rice (*Oryza sativa*) caused by *Xanthomonas oryzae* pv. *oryzae* (Xoo), a member of the γ-proteobacteria, is one of the most serious rice pathogens worldwide [1,2]. Xoo invades rice xylem tissue through water or wounds resulting in systemic infection [3]. Planting resistant lines is the most economical and effective way to control this vascular disease [4]. To date, 38 bacterial-blight resistance genes have been reported in rice, eight of which are from wild species [5]: *Xa10* (*O*. *Cas209*) [6,7], *Xa21* (*O*. *longistaminata*) [8], *Xa23* (*O*. *rufipogon*) [9], *Xa27* (*O*. *minuta*) [10], *Xa29* (*O*. *officinalis*) [11], *Xa30* (*O*. *rufipogon*) [12], *Xa32* (*O*. *australiensis*) [13] and *xa*32 (*O*. *meyeriana*) [14].

*O*. *meyeriana* has strong resistance to all Xoo strains [15] but no resistance gene has yet been cloned from it [14]. There is hybrid sterility between *O*. *meyeriana* and cultivated rice [16] but the BB resistance of *O*. *meyeriana* has been introduced into cultivated rice by asymmetric somatic hybridization [16,17] leading to the development of some highly resistant rice lines including Y73 [18] and SH76 [19]. Some insights have been obtained into these highly resistant phenotypes by a microarray analysis to examine transcription in Y73 and a proteomics study of SH76. 115 genes had altered RNA expression in Y73 in response to Xoo, and they involved in oxidant redox, signal transduction and transcription. Seven of them were up- regulated more than fivefold, including two transcription factors (TFs) and one ubiquitination protein [19]. 34 proteins changed significantly in concentration in response to Xoo in SH76, and they relate to signal transduction, photosynthesis, antioxidant defense and metabolism [19]. A small auxin RNA was up in Y73 [18], and a auxin–regualted protein up regulated in SH76. Besides, a Rubisco Large subunit (RcbL) was degraded [19]. Rubisco activity is regulated by Rubisco activase (RCA). RCA moved to the thylakoid membrane in *O*. *meyeriana* 12h to 16h after inoculation with Xoo coupled with an oxidative burst, while RCA remained in chloroplast stroma in the susceptible line [20].

Secreted proteins play an important role in the rice-Xoo interaction [21–23], and studies of the rice secretome have identified some proteins in the plasma membrane [24], xylem sap [25] and leaves [26] that are involved in the early defense responses to Xoo. Cell suspension cultures have been used to study the secretome of many plants, including *Arabidopsis thaliana* [27], maize [28], tobacco [29], medicago [30] and rice [31] but there have been no reported studies using this method to investigate changes in the secretome of resistant rice in response to Xoo infection.

Here, we used two-dimensional difference gel electrophoresis (2D-DIGE) coupled with Mass Spectrometry (MS) to study secretome changes in an *O*. *meyeriana* embryo cell suspension in response to inoculation with Xoo. A total of 34 differentially expressed proteins were identified and their possible roles in response to Xoo are discussed.

## 2 Materials and Methods

### 2.1 Plant material

Sterile *Oryza meyriana* seedlings were cut into 1–1.5 cm pieces and placed on MS callus induction medium (3 mg/L 2,4-D, 0.3 mg/L 6-BA) [18]. After incubation in the dark at 28°C for 2 wk, they were transferred to a 16h/8h-light/dark regime at 28°C for 3 wk to induce calli. Growing calli (0.5–1.0g) were transferred into liquid MS medium (2.5 mg/L 2,4-D, 0.3 mg/L 6-BA) and shaken (150 rpm) at 28°C in the dark [32]. The suspension culture was sub-cultured weekly until the cells appeared dense, uniform and light yellow. Xoo strain PXO124 (race P10) was cultured on PSA liquid medium (1% tryptone, 0.1% yeast extract, 1% sucrose, 0.3% peptone, 1.5% agar) at 28°C for 48 h and adjusted to 10^8^ CFU ml^-1^ before inoculation to the rice suspension culture 3 days after sub-culturing. For qQRT-PCR and peroxidase activity assays, leaves from 21 day old *O*. *meyeriana* and Nipponbare (susceptible control) seedlings were inoculated with Xoo (OD ~0.8) using the leaf-cutting method [33].

### 2.2 Isolation secreted and total proteins from *Oryza meyriana* suspension—cultured cells

After sub-culturing for three days, the rice culture medium was harvested 0 h and 24 h after inoculation with Xoo. Calli were collected through a filter (0.18 mm), washed with ddH_2_O and stored at -80°C. The filtered medium was centrifuged at 20,000 g for 20 min to remove the residual cells, and the supernatant was concentrated using Sartorius Slice 200 (Sartorius, Germany). Secreted and total proteins were extracted by a modified phenol-methanol method [34]. Four biological replicates were prepared. Secreted protein and total protein were further purified using the 2-D Clean Up Kit (GE healthcare, USA) and their concentrations determined using a 2-D Quant kit (GE healthcare, USA).

### 2.3 Western blot analysis

Approximately 15 μg of secreted proteins (S) and total proteins (T) were separated on a 12.5% (w/v) SDS-PAGE gel overlaid with a 4% (w/v) sticking gel, and electrotransferred onto a PVDF membrane. After blocking in blocking buffer [20 mM Tris-HCl, pH 7.6, 0.5 mM NaCl, 0.005% v/v Tween 20, 1% (w/v) BSA] overnight, the membrane was incubated with mouse anti-actin antibody (1:1000, Abmart, Shanghai, China) followed by the secondary antibody, mouse anti-mouse IgG conjugated with alkaline phosphatase (1:10000, Sigma, Missouri, USA). Binding of the enzyme conjugated antibodies was detected with the NBI/BCIP (Amresco, Ohio, USA) system. For western blotting of the peroxidase, extracts were prepared from 0.1 g leaves of *O*. *meyeriana* and cultivated rice Nipponbare (susceptible control) at 0 h (mock-control) and 24 h post-inoculation with Xoo. They were homogenized in 1 ml Extract Buffer [20mM Tris-HCl, 200mM NaCl, 1mM EDTA, 1mM DTT, β-mercaptoethanol (10%, v/v)], samples of approximately 10 μl (~4.5 μg/μl) were separated on SDS-PAGE gels and the blot membrane was incubated with rabbit anti- peroxidase antibody (1:10,000, Agrisera, Sweden).

### 2.4 2D-DIGE, Image Scanning and Analysis

Prepared secreted proteins were separated by 2D-DIGE as described previously [34]. The Cy2, Cy3 and Cy5 labeled samples (50 μg) were mixed and loaded on the strips (linear, 24cm, pI 4–7, GE Healthcare, USA) for the first dimension separation. Next, the strips were placed on top of 12.5% SDS-PAGE gels for the second dimension electrophoresis. Protein spots on gels were scanned using an Ettan DIGE Scanner (GE Healthcare, USA) and the images were analyzed using Decyder 2D software (Version 7.0, GE Healthcare, USA). Finally, spots from different gels were matched using Biological Variation Analysis. Only spots present in all gels and which exhibited statistically significant changes in intensity (≥1.5 fold or ≤-1.5 fold, p <0.05) were considered to be differentially expressed proteins.

### 2.5 In gel protein digestion and MS analysis

About 500 μg secreted proteins were loaded on the strips, separated by 2-DE and stained with Coomasie Brilliant Blue (CBB) R-250. Differentially expressed protein spots were manually excised from the stained 2-D gel and transferred to a sterilized tube (1.5 ml) with destaining solution [30% (w/v) acetonitrile (ACN), 100 mmol NH_4_HCO_3_]. After being vacuum freeze-dried, the spots were digested in 30 μl enzyme buffer (50 mmol NH_4_HCO_3_, 50 ng/μl trypsin) at 37°C overnight. Then, the small peptides were back-extracted in 60% ACN buffer [0.1% trifluoroacetic acid (TFA)] and dried under a stream of nitrogen. Finally, peptides were re-suspended in 50% ACN buffer [0.1% w/v TFA, 5 mg/ml α- cyano -4-hydroxycinnamic acid (CHCA)], analyzed using an ABI 4800 Proteomics Analyzer MALDI-TOF/TOF (Applied Biosystems, Foster City), and MALDI-TOF spectra were searched. All MALDI-TOF spectra were searched against the National Center for Biotechnology Information non-redundant (NCBInr) database using the GPS Explorer^TM^ software (v3.6, Applied Biosystems) and MASCOT search program (v2.1 Matrix Science). Finally, based on the MALDI-TOF-MS, only protein scores > 95 (p <0.05) were accepted for the identification of protein samples.

### 2.6 Bioinformatics analysis

Homologues of the identified proteins were searched in the RAGP (http://rice.plantbiology.msu.edu/) for matching against the NCBI database. The UniProt (http://www.uniprot.org/) database was used to determine their functions. Next, SignalP (version 4.1, http://www.cbs.dtu.dk/services/SignalP/) was used to predict their secretion pathways and PSORT II (http://psort.hgc.jp/form2.html) to predict their subcellular location. The identified proteins were annotated and grouped according to their predicted function and biological processes.

### 2.7 RNA extraction and Quantitative real time PCR for gene transcriptional expression analysis

Primers were designed using primer 6 (version 6.0), to be specific to the corresponding gene sequences of the proteins identified by MS in the RAGP database (S1 and S2 Tables). Total RNAs were extracted from inoculated leaves of *O*. *meyeriana* and Nipponbare (4–5 leaf stage) using TRizol reagent (Invitrogen, Germany). Residual DNA was removed by RNase-free DNase (Fermentas, Canada). The first strand cDNAs were synthesized by Synthesis kit for qRT-PCR (Bio-RAD, USA). qRT-PCR with Eva Green (Biotium, USA) was performed on the light Cycler 480 real-time PCR system (Roche, Basle, Switzerland), and the PCR program consisted of: 95°C for 3 min, 45 cycles of 95°C for 30s, 55°C for 30s and 72°C for 30s. Dissociation curves were generated at the end of PCR cycles, and relative gene expression was calculated by the 2^- Ä (Ä Cp)^ method. The reference gene was OsActin gene (AK 060893), and all experiments were repeated three times with cDNA prepared from different biological samples.

### 2.8 POD activity detection

POD activity was detected in *O*. *meyeriana* and 21-day-old Nipponbare seedling leaves at 0h, 6h, 12h and 24h after inoculation with Xoo. Peroxidase activity was assayed as described in the POD detection kit (Keming, China) with some modifications. 0.1 g rice leaves were homogenized in 1ml cold extraction buffer, and centrifuged at 8,000g for 10 min at 4°C. 3.75 ul samples were mixed with 263.75 ul reaction buffer and the absorbance was measured by Spectra Max M5 (Molecular Devices, USA) at 470 nm for 30s (A1) and 60s (A2). Calculation of POD was based on the following formula: POD(U/g) = 7133×(A2-A1)÷Sample weight. One enzyme unit is defined as the absorbance of 1g sample in reaction buffer at 470 nm for a 60s change of 0.01. All experiments were repeated three times prepared from different biological samples

## 3 Results

### 3.1 Western blot analysis indicates that the secreted proteins preparation may be free from contamination with non- secreted proteins

**Fig 1:**
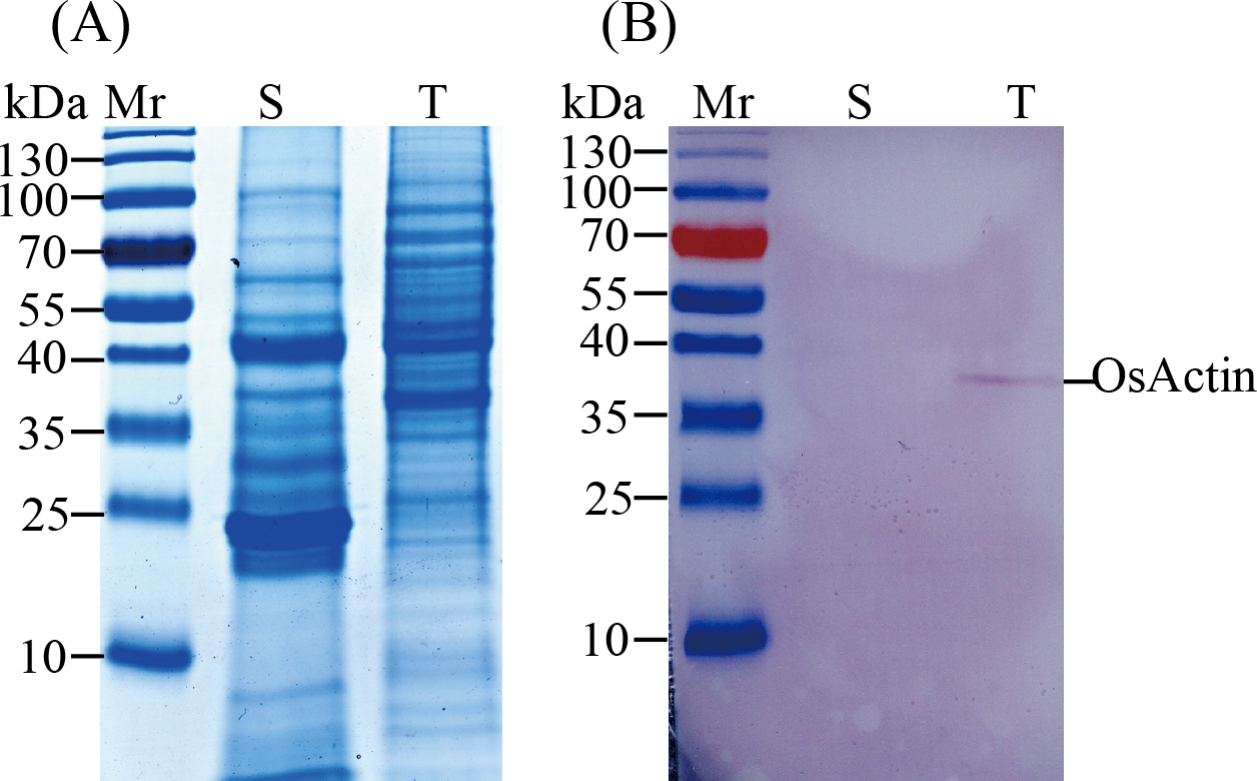
Western blot of the secretome and total proteins from *Oryza meyeriana*. A: Secretome and total protein were separated by 1D SDS-PAGE; B: Secretome and total protein were analyzed by immunoblotting with a mouse anti-actin antibody. Samples of 15 μg protein were loaded onto 12% SDS-PAGE gels, and stained with Coomassie Brilliant Blue (CBB) R-250 solution. For western blot, actin was used a representative intracellular protein. Mr: marker; S: secretome; T: total protein.

Preparation of secreted proteins is a challenging work [31]. Previously, we tested four protein isolation methods, including TCA/acetone [20], molecular weight cutoff filters, Ammonium sulfate precipitation and the phenol method [34] and found that the phenol method was suitable for extracting and isolating secreted proteins from *O*. *meyeriana* suspension culture. In this study, Western blot showed that actin (45 kDa) was detected only in the total proteins, but was absent from the secreted proteins (Fig 1). This suggests that the secreted proteins prepared from suspension culture may be free from contamination with non-secreted proteins.

### 3.2 Identification of differentially expressed proteins

**Fig 2:**
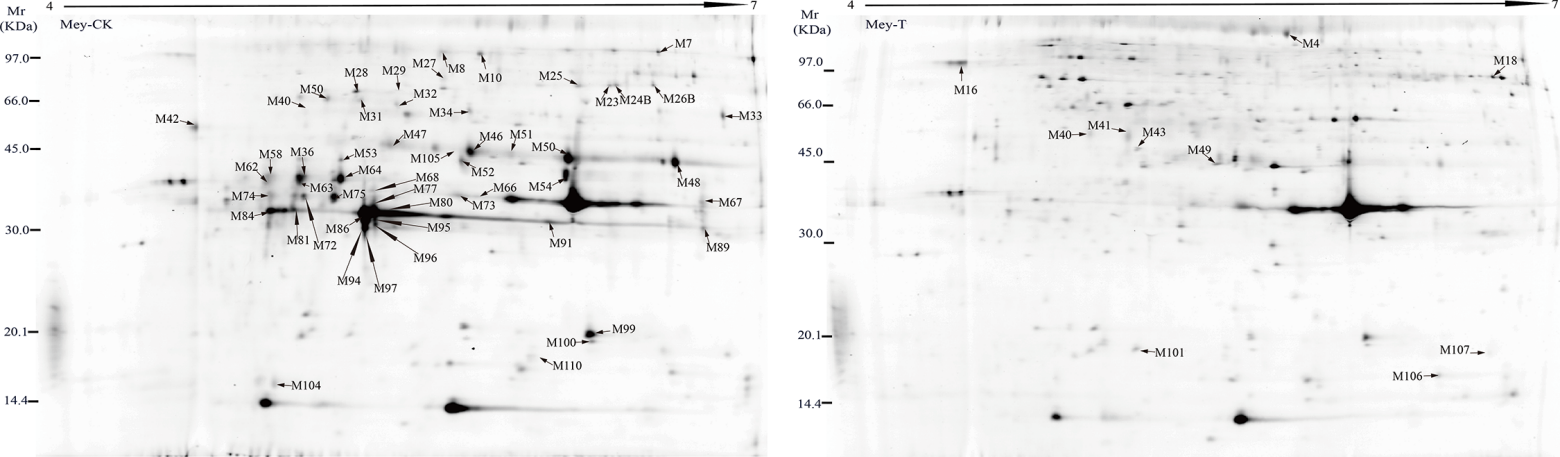
2D-DIGE analysis of the Xoo-induced secretome in *O*.*meyeriana* suspension cultured medium. Secreted-proteins were extracted from the medium and separated by 2D-DIGE using pH 4-7/24cm linear IPG strips and 12.5% SDS-PAGE gels. 110 protein spots showed significant changes (> 1.5 fold; p< 0.05), corresponding to 34 proteins identified by MS.

Secreted proteins from *O*. *meyriana* suspension culture were isolated using the phenol method, separated by 2D-DIGE and identified by MS. More than 1500 protein spots were detected reproducibly on gels by DeCyder 2D software of which 110 changed in intensity significantly (p < 0.05) and by more than 1.5 fold when compared with the control (Fig 2). After analysis by MS, 72 proteins corresponding to 34 genes were matched to the NCBI database. Among the identified proteins, 10 were up regulated and 24 down regulated (Table 1).

**Table 1:** Differential proteins identified by MS.

No^a^	Accession No.	Locus	Unique Peptide^b^	SC ^c^ %	Average fold change^d^	Molecular function and/or property	SignalP^e^	Cell location
**Signal transduction**								
M18	gi|326515056	Os12g44020.1	3	10	1.73±0.016	Ser/Thr protein phosphatase family protein, expressed	-	extracellular
M30	gi|115477980	Os09g02729.1	6	15	-1.78±0.0058	phospholipase C, expressed	Y	extracellular
M43	gi|357128757	Os05g44200.1	7	15	2.37±0.045	GDSL-like lipase/acyl hydrolase, expressed	Y	extracellular
M76	gi|115483362	Os08g42580.1	11	29	1.71±0.034	protein kinase domain containing protein, expressed	-	cytoplasmic
**Protein metabolism**								
M11	gi|315797642	Os12g17910	4	26	12.98±0.042	T-complex protein, expressed	-	ER
M55	gi|149392557	Os05g24550.1	7	20	-3.77±0.00017	Papain family cysteine protease domain containing protein, expressed	-	cytoplasmic
M101	gi|62546209	Os11g09280.1/2	9	13	1.63±0.035	OsPDIL1-1 protein disulfide isomerase PDIL1-1, expressed	Y	ER
**PRs and defense proteins**								
M8	gi|62733152	Os11g47820.2	8	25	-1.64±0.039	glucan endo-1,3-beta-glucosidase	Y	mitochondrial
M16	gi|242032727	Os03g57880.3	6	12	3.52±0.0052	glucan endo-1,3-beta-glucosidase, expressed	Y	extracellular
M40	gi|116309669	Os04g51460.1	9	28	3.54±0.000027	glycosyl hydrolases family 16, expressed	Y	extracellular
M41	gi|218195513	Os04g51460.1	8	24	3.58±0.00067	glycosyl hydrolases family 16, expressed	Y	extracellular
M42	gi|49387539	Os02g42310.1	5	12	-1.69±0.023	OsSCP8—Putative Serine Carboxypeptidase homologue, expressed	-	extracellular
M49	gi|125560666	Os08g13920.1	7	25	2.57±0.0051	glycosyl hydrolases family 16, expressed	Y	cytoplasmic
M66	gi|151935395	Os05g31140.3	2	14	-1.85±0.003	glycosyl hydrolases family 17, expressed	-	extracellular
M28	gi|125526903	Os01g43490.1	4	12	-5.96±0.0011	polygalacturonase, expressed	Y	cytoplasmic
M90	gi|304301588	Os07g04560	1	12	-9.82±0.025	no apical meristem protein, expressed	-	mitochondrial
M106	gi|115452789	Os03g21040.2	11	27	1.53±0.048	stress responsive protein, expressed	Y	cytoplasmic
M107	gi|125586117	Os03g21040.2	12	31	1.61±0.025	stress responsive protein, expressed	Y	cytoplasmic
**Redox**								
M4	gi|293336711	Os03g57880.3	5	12	2.07±0.036	monocopper oxidase, expressed	Y	extracellular
M31	gi|55700999	Os04g51460.1	2	7	-2.57±0.0028	Peroxidase expressed	Y	extracellular
M32	gi|242089641	Os04g51460.1	6	14	-1.75±0.0047	Peroxidase expressed	Y	mitochondrial
M33	gi|115477493	Os02g42310.1	8	25	-5.13±0.0011	hypothetical protein		ER
M34	gi|45685281	Os08g13920.1	6	16	-1.72±0.032	peroxidase expressed	Y	extracellular
M52	gi|55701087	Os05g31140.3	4	10	-2.2±0.000038	peroxidase expressed	Y	extracellular
M99	gi|115473931	Os01g43490.1	6	40	-1.64±0.049	copper/zinc superoxide dismutase, expressed	N	cytoplasmic
M105	gi|115474057	Os07g04560	4	14	-2.03±0.0019	hypothetical protein	Y	extracellular
M111	gi|357473921	Os03g21040.2	3	15	-1.93±0.0045	peroxidase expressed	Y	nuclear
**Cell wall structure proteins**								
M23	gi|125524938	Os01g12070.1	6	9	-2.67±0.0062	endoglucanase, expressed	Y	ER
M10	gi|326491047	Os11g05760.1	4	13	7.34±0.0069	OsProCP5—Putative Lysosomal Pro-x Carboxypeptidase homologue, expressed	Y	mitochondrial
M27	gi|57899795	Os01g66830.1	4	13	-1.71±0.007	pectin acetylesterase domain containing protein, expressed	Y	Golgi
M78	gi|162458364	Os10g40720.1	7	12	-1.75±0.0019	expansin expressed	-	vesicles of secretory system
M89	gi|125532912	Os10g40700.1	9	32	-7.12±0.00083	Expansine expressed	Y	extracellular
M94	gi|115462831	Os05g15690.1	2	13	-32.47±0.001	expansin expressed	Y	extracellular
M95	gi|116310398	Os04g46630.1	1	5	-38.52±0.0002	expansin expressed	Y	cytoplasmic
M96	gi|125551519	Os05g15690.1	1	10	-32.99±0.00059	expansin expressed	Y	extracellular
M97	gi|125549272	Os04g46630.1	3	17	-13.57±0.000058	expansin expressed	Y	cytoplasmic
M104	gi|115483362	Os10g40720.1	10	28	-5.55±0.0034	expansin expressed	Y	extracellular

^a^ spot number as given in Fig 2. Some proteins from different spots correspond to the same gene suggesting they may have been post-translationally modified.

^b^ number of matched peptides

^c^ sequence coverage

^d^ Fold change with p <0.05

^e^ “Yes” means protein was predicted to have a signal peptide.

### 3.3 Bioinformatics analysis of differentially expressed proteins

Thirty four proteins from rice were identified by BLAST in the RAGP and Uniprot database, and classified into five functional groups (Table 1). These include proteins with signaling roles, proteins involved in redox and defense, cell wall proteins and proteins involved in metabolism. Twenty six proteins were predicted to have signal peptides and more than 17 were predicted to be located in the extracellular space. Three proteins without signal peptides (M18, M42, M78) were predicted to be located in the extracellular space. Proteins can be transported across membranes through the classical or non classical secretory pathways [35]. For example, the key gluconeogenic enzyme in the Calvin Cycle, fructose-1,6-bisphosphatase [36] does not contain signal sequences but is secreted via a non-classical secretory pathway similar to our results.

### 3.4 Correlation between protein and mRNA expression

**Fig 3:**
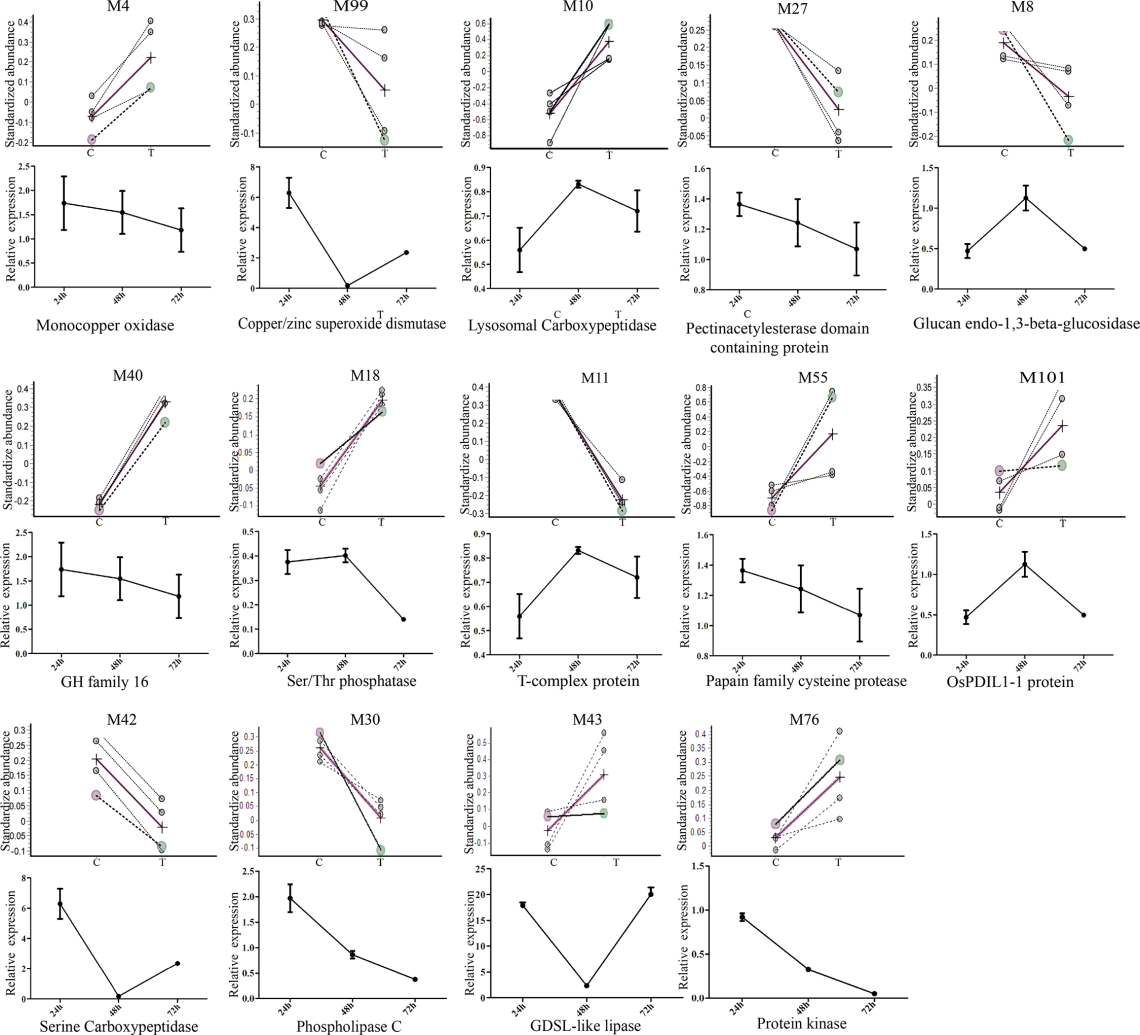
Xoo-responsive rice proteins identified by 2-D DIGE. Changes in protein abundance of the 14 rice proteins in the secreted protein fraction of suspension-cultured cells 24 h after inoculation (top panel); mRNA expression of the 14 rice genes in the leaves of rice seedlings 24 h, 48 h and 72 h after inoculation (bottom panel).

To investigate the relationship between the identified proteins and their transcription, Quantitative real time PCR was performed to analyze the transcriptional activities of 14 randomly selected rice genes corresponding to the rice proteins identified. Samples were tested at 0 h, 24 h, 48 h, and 72 h post inoculation with Xoo. Three up-and three down regulated proteins showed good correlation between protein and RNA expression at 24 hpi, including a monocopper oxidase, a GDSL-like lipase, a pectinacetylesterase, a glucan endo-1,3-beta-glucosidase, a protein kinase and glycosyl hydrolases (Fig 3). Transcription of the remaining eight proteins was not well correlated with their protein expression at 24 hpi. The mRNA transcriptional level does not always correlate well with the protein expression levels [37]. For example, it was reported that Phospholipase C was down regulated, but the transcription was up regulated [38], similar to our results. The transcription of many chilling- related proteins was first up and then down regulated after cold stress [39]. It should be noted that suspension cultured cells were used to measure secreted protein expression levels, while transcript levels were determined from seedling leaves. In addition, gene expression in infected leaves may also be influenced by the circadian rhythm.

### 3.5 Quantitative Real Time PCR and Enzyme activity assay for Peroxidases

**Fig 4:**
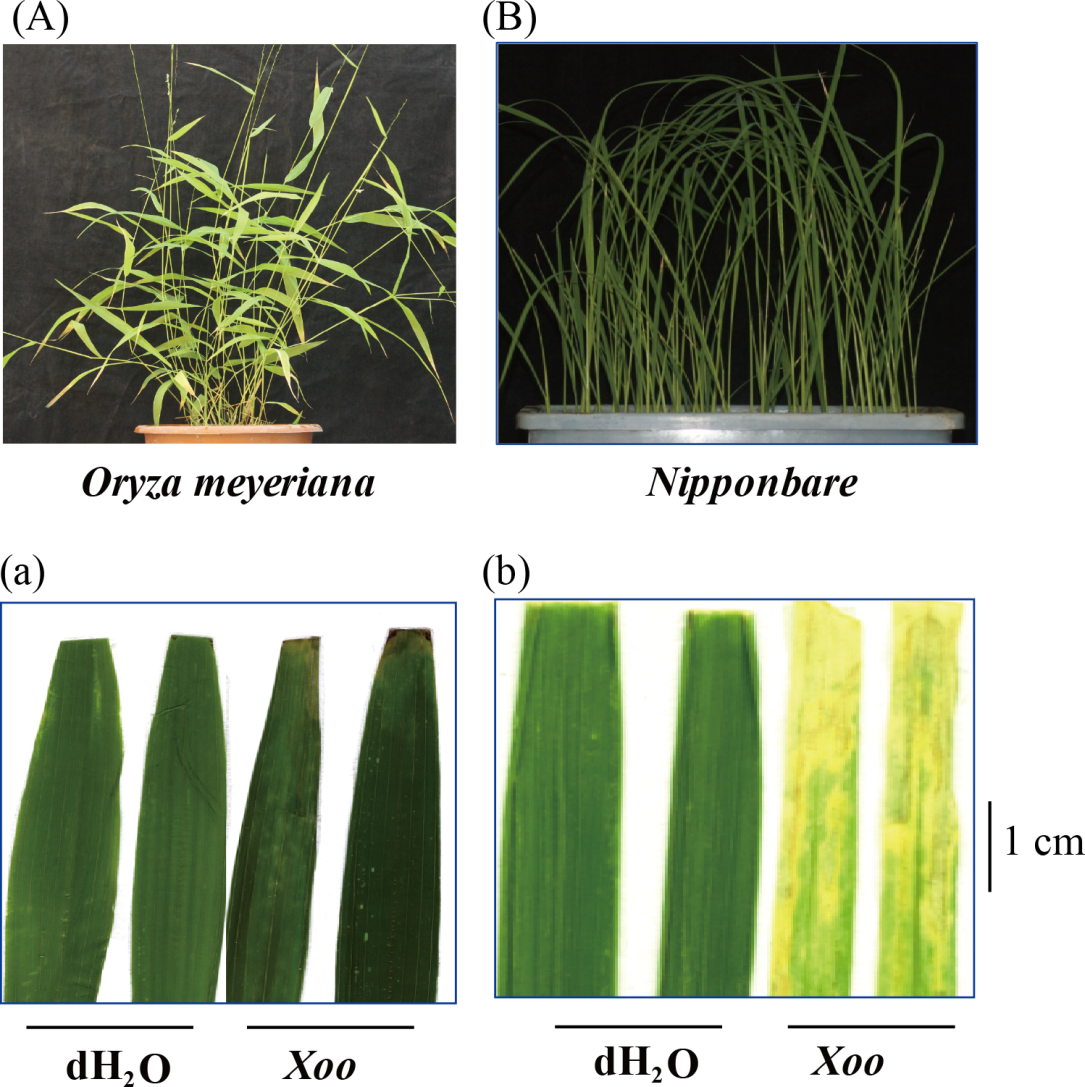
The effect of Xoo (PXO124) on *O*. *meyeriana* and susceptible cultivated rice Nipponbare. Upper panels show the plant phenotypes; lower panels show leaves 14 days after inoculation with Xoo using the clipping method. Controls were inoculated with water.

**Fig 5:**
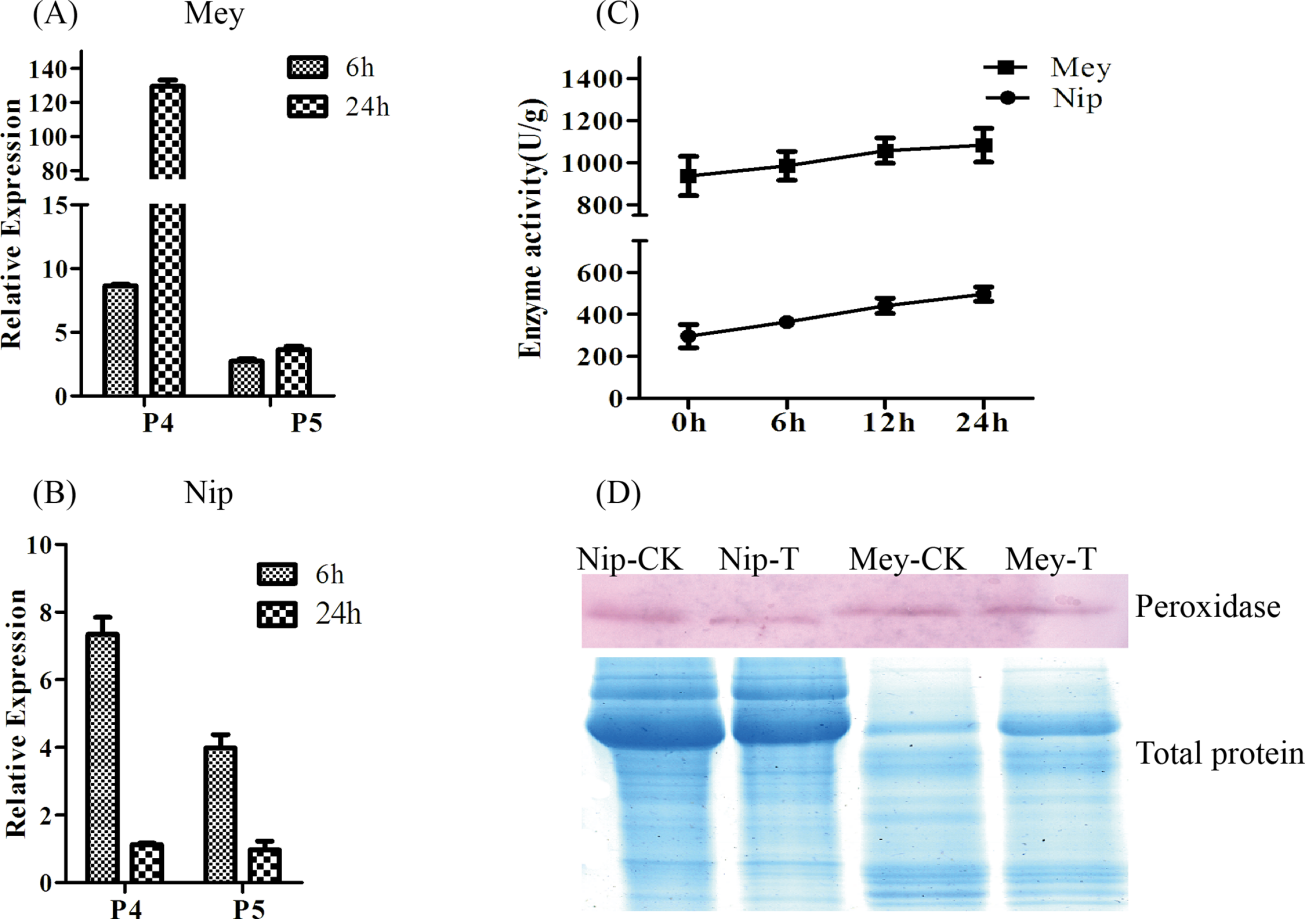
qQRT-PCR and enzyme activity assay of peroxidases in *O*. *meyeriana* and Nipponbare. (A and B) qQRT-PCR of peroxidase 4 (P4) and peroxidase 5 (P5) in *O*. *meyeriana* (A) or Nipponbare (B) 6h and 24h after inoculation with Xoo. (C) Peroxidase activity 0h, 6h, 12h and 24h after inoculation with Xoo; OsActin was used as a mock gene. (D) Western blot of peroxidase in *O*. *meyeriana* and Nipponbare 0h and 24h after inoculation with Xoo (top panel); total protein stained with CBB (bottom panel). CK: Control Mock; T: Treated with Xoo.

Many peroxidase proteins were down regulated in *O*. *meyeriana* (Table 1). Quantitative Real Time PCR was performed to analyze the transcriptional activities of two randomly selected peroxidase genes, corresponding to the rice proteins identified (P4 and P5; for primers see S2 Table). Cultivated rice Nipponbare is high susceptible to Xoo (Fig 4). The transcription of P4 and P5 was up regulated in *O*. *meyeriana* in response to Xoo, while they were both down regulated in Nipponbare (Fig 5A and 5B). Peroxidase enzyme activity was up regulated in both *O*. *meyeriana* and Nipponbare, with the activity in *O*. *meyeriana* consistently about three times higher than in Nipponbare (Fig 5C). Expression of the peroxidase proteins was slightly down regulated in Nipponbare, consistent with their transcription, but they were unchanged in *O*. *meyeriana* (Fig 5D).

## 4 Discussion

Secreted proteins play important roles in a number of pathological processes, such as signal transduction [40] and defense [41]. Suspension-culture is a model system for studying the secreted proteins [42] and has been widely used for secretome analysis of interactions between plants and their pathogens including bacteria [43] and fungi [44]. In this study, we used this method to investigate the altered protein expression in resistant rice cells in response to *Xoo* inoculation.

### 4.1 Signal transduction secreted proteins

Signal transduction plays important roles in many signal networks in activating the plant immune response [45]. GDSL has a crucial role in signal transduction [46] and can enhance resistance through the ethylene signaling pathway [47]. Overexpression of GDSL can increase disease resistance and abiotic stress tolerance [48,49]. Ser/Thr protein phosphatases (PP2C) can negatively regulate the snRK2 signal pathway by terminating signal transduction cascades [50]. Lysin motif (LysM) receptor-like kinase is a cell membrane protein that can enhance host disease resistance through the LysM signaling pathway [51]. Here, we observed that a GDSL-like lipase (Spot M43), a PP2C (Spot M18) and a LysM receptor-like kinase (Spot M76) were up regulated in *O*. *meyeriana* suspension culture in response to Xoo inoculation. Phospholipase C (Spot M30) and carboxypeptidase D (Spot M44) were both down regulated. Plant resistance to pathogens is very complex and may include recognition, signal transduction and activation of host defense systems [52]. Further investigation is needed to determine whether the three up regulated signal proteins identified in this study are the main reason for the resistance of *O*. *meyeriana* to Xoo.

### 4.2 Defense proteins

Systemic induction of pathogenesis-related (PR) proteins is important in defense against plant pathogens [53]. Glucan endo-1,3-beta glucosidase, Glycoside hydrolase (GH) 16 and GH17 all belong to the OsPR2 family. Glucan endo- 1,3-beta glucosidase can inhibit the growth of fungi by degrading their cell walls [54]. GH proteins are important enzymes in chemical defense [55]. Here, we observed a Glucan endo-1,3-beta glucosidase (Spot M8) and a GH16 (M40) protein that were up- regulated under the stress of Xoo infection, while a GH17 (M66) protein and a polygalacturonase (M68) were down regulated. GH 45 was reported to be down regulated in response to a pathogen [56] similar to our results. GH16 may be involved in chemical defense and Glucan endo-1,3-beta glucosidase may be involved in degrading the cell wall [57]. Further experimental work is needed to determine whether these two up regulated OsPR2 proteins make it more difficult for Xoo to infect cells and thus strengthen host resistance.

### 4.3 Cell wall structural proteins

The plant cell wall plays an important role as the first defensive barrier against pathogen invasion [58–60]. Expansins are cell wall glycoproteins that accumulate more quickly in resistant than in susceptible cotton after inoculation with its pathogen [61,62]. However, indole-3-acetic acid-amino synthetase (GH3–8) can inhibit the expression of Expansins and enhance rice resistance to *Xoo* [63]. Pectin acetylesterase can degrade pectin and soften the primary cell wall [64]. Here, we observed that several Expansins and one pectin acetylesterase were down regulated in *O*. *meyeriana* suspension culture in response to Xoo infection. It would be interesting to investigate whether the down regulated Expansins are inhibited by GH3-8 that activates the immune response through the Salicylate-(SA) and Jasmonate (JA) signaling pathway.

### 4.4 Oxidative stress proteins

It is well known that an oxidative burst is one of the first responses of plants when attacked by pathogens, and that it is always accompanied by activation of PR genes [65]. ROS is a signaling molecule that is involved in triggering the hypersensitive response (HR) inducing systemic acquired resistance, but also has roles in many other processes including PCD (programmed cell death) and in responses to biotic and abiotic stress [66]. However, regulation of ROS is extremely complex. In Arabidopsis, at least 152 genes form a complex gene network controlling ROS toxicity [67]. Peroxidases play crucial roles in regulating ROS [68] and extracellular peroxidases (POXs) can be induced by JA, SA, ABA, thus participating in plant defense [69]. In our results, peroxidase was down regulated but its transcription was up regulated similar to a previous report [38]. Peroxidase enzyme activity was up regulated in *O*. *meyeriana* in response to Xoo infection. Enzyme activity can be regulated by other factors, such as hormones and metal ions. Hydrogen peroxide can rapidly accumulate in mesophyll cell gaps in the early stages of Xoo infection [70]. Expression of peroxidase and HR happens simultaneously during pathogen invasion [71]. We suggest that there may be a rapid and dramatic HR induced by peroxidase in *O*. *meyeriana* during the early stages of Xoo invasion that needs to be further studied.

### 4.5 Comparison with experiment using susceptible rice

Plants possess a complex defense mechanisms to fend off infection by pathogens [72], and the host resistance is determined by the genotypes of both the plant and its pathogen [73]. Host responses to their pathogens are classified into two groups: Incompatible (resistance) and Compatible (susceptibility) [74]. In previous work, we used proteomics to analyze the secreted proteins of a susceptible rice suspension culture challenged by Xoo [75] and comparisons with this study show a different pattern of secreted proteins (S1 Fig). In susceptible rice culture, seven rice proteins responded to Xoo and four Xoo proteins also changed in intensity. Three of the Xoo proteins were localized in the cell membrane and one protein, Xoo3654, is likely a negative regulator of Xoo virulence. In contrast, we did not detect any Xoo proteins in the *O*. *meyeriana* suspension culture. The seven proteins that responded to Xoo in susceptible rice are involved in cell wall modification, the TCA cycle, glycolysis and redox. Many more proteins involved in redox and cell wall modification were detected in *O*. *meyeriana* along with expansins (which were not detected in susceptible rice). These results suggest that the defense mechanism of *O*. *meyeriana* is not merely an amplification of the response of susceptible rice.

## Concluding Remarks

This study has established a secreted protein extraction and purification procedure which can enrich the lower-concentration secreted protein from the suspension cultures. We used 2D-DIGE coupled with MS to analyze differentially expressed secreted proteins in resistant rice suspension culture in response to Xoo infection. The identified proteins are involved in various biological processes, including signal transduction, defense, reactive oxygen species and cell wall modification. We observed that peroxidase activity in *O*. *meyeriana* is about three time higher than that in susceptible rice. Together, those results not only help us better understand the interaction between resistance rice and Xoo, but also serve as a reference for studying the interaction between other plants and their pathogens.

## Supporting Information

S1 Fig Classification of *Xoo*-responsive proteins in *O*. *meyeriana* and susceptible rice. A: classification of *Xoo*-responsive proteins in Meyeriana; B: *Xoo*- responsive proteins in Nipponbare (Susceptible rice) [73]. ↑: up regulated protein; ↓: down regulated protein.
(PDF)

S1 Table Primers used for QRT-PCR to detect the mRNA of secreted proteins from *Oryza meyeriana*. (PDF)

S2 Table Primers used for QRT-PCR to detect the mRNA of peroxidases of *Oryza meyeriana*. (PDF)
