# Supplementary material for: Analysis of the Proteins Secreted from the Oryza meyeriana Suspension-Cultured Cells Induced by Xanthomonas oryzae pv. oryzae
Source: PLoS One. 2016 May 19;11(5):e0154793. doi: 10.1371/journal.pone.0154793 (PMC4873123; doi:10.1371/journal.pone.0154793)
Supplement: S1 Fig — A: classification of Xoo-responsive proteins in Meyeriana; B: Xoo- responsive proteins in Nipponbare (Susceptible rice) [73]. ↑: up regulated protein; ↓: down regulated protein. (PDF) [file pone.0154793.s001.pdf]

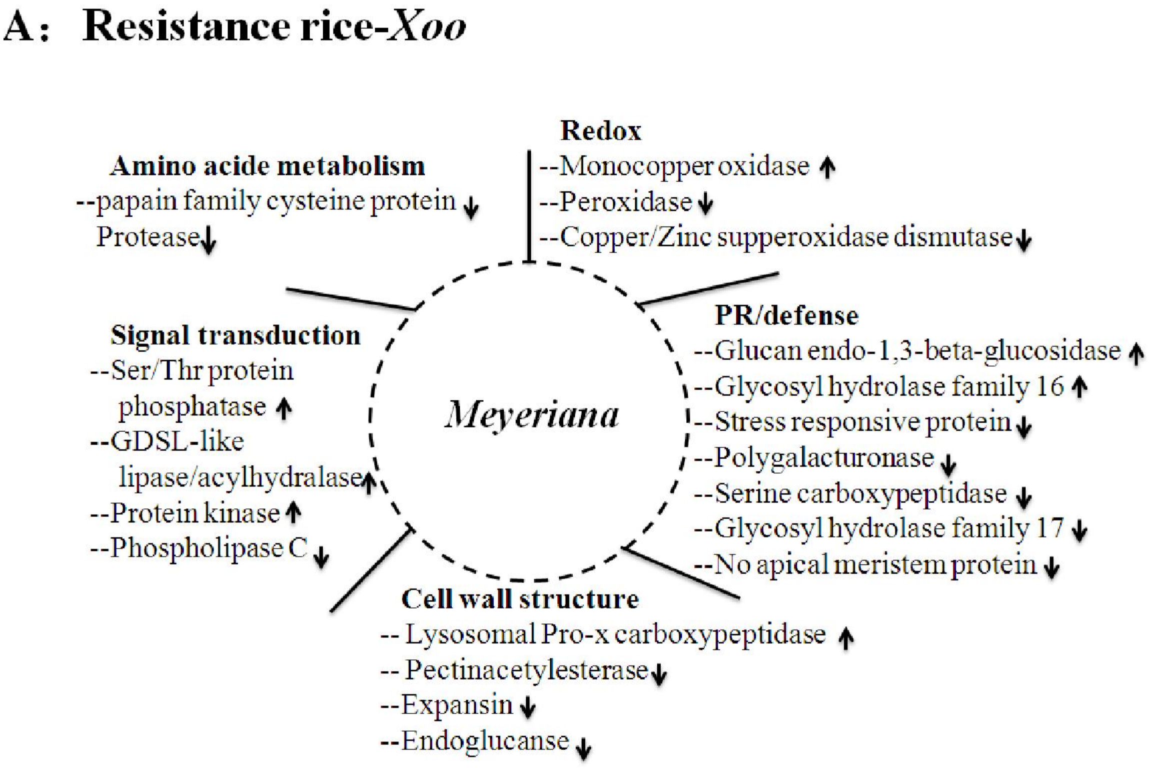

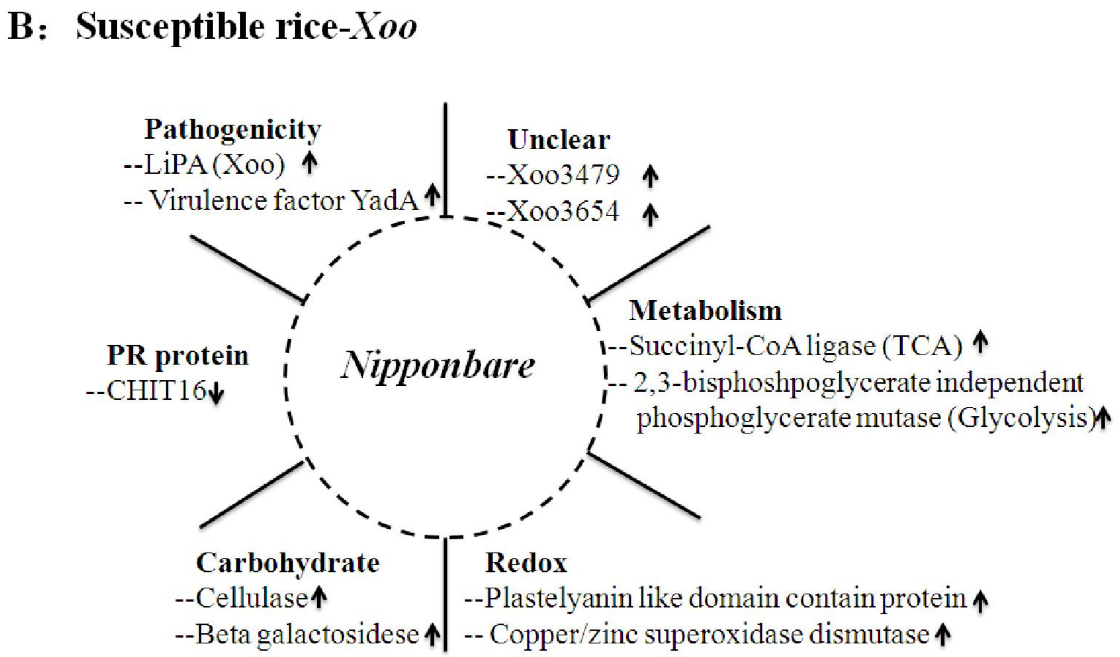

S1 Fig. Classification of *Xoo*-responsive proteins in *O. meyeriana* and susceptible rice. A: classification of *Xoo*-responsive proteins in Meyeriana; B: *Xoo*- responsive proteins in Nipponbare (Susceptible rice) [73]. ↑: up regulated protein; ↓:down regulated protein.
