# Supplementary material for: Analysis of the Proteins Secreted from the Oryza meyeriana Suspension-Cultured Cells Induced by Xanthomonas oryzae pv. oryzae
Source: PLoS One. 2016 May 19;11(5):e0154793. doi: 10.1371/journal.pone.0154793 (PMC4873123; doi:10.1371/journal.pone.0154793)
Supplement: S1 Table — (PDF) [file pone.0154793.s002.pdf]

S1 Table. Primers used for QRT-PCR to detect the mRNA of secreted proteins from *Oryza meyeriana*

Spot No	NCBI Accession number	Sequence (5'-3')	Product size (bp)
**4**	Os06g01490.1	F: GATGGAGGCTATAATGTCACT	**123**
		R: CAGAGGATGATGAACTTGATG	
**8**	Os11g47820.2	F: CCGAATCCATCACCTAACC	**131**
		R: CAGTCCACATAGCCACAG	
**10**	Os11g05760.1	F: CGGCGGAATACTGAAGAG	**139**
		R: GATTCTCATCTCCTGTCTCC	
**11**	Os12g17910	F: ACAATGCTGGAGTGGAAG	**108**
		R: CCGCCTCAATCAAGTTCT	
**18**	Os12g44020.1	F: GTTCTGGTACTCCGTCAAG	**189**
		R: TAGTGGTAGTTGTTGCTGTT	
**27**	Os01g66830.1	F: ACGCCATTCAACATCTCAT	**134**
		R: TTACAAGGATACTCGCAGTC	
**30**	Os09g02729.1	F: TGTAGAGAATGAAGCGTGT	**198**
		R: CAAGCAGCAATGGTGTTG	
**40**	Os04g51460.1	F: GGCAAAGAGCAGCAGTTC	**129**
		R: GCGTGGTTCTTCATCTCC	
**42**	Os02g42310.1	F: ATCAGCAGGTTGGAGGAT	**148**
		R: GTCGGAGGCATCTTCTTG	
**43**	Os05g44200.1	F: CTTCACCTACATCAACATCTAC	**117**
		R: ACCTGACCGTTGTTCCTC	
**55**	Os05g24550.1	F: GGATGCGAACCTGCTTAT	**141**
		R: GTAGACCTCTGCCATTATGT	
**76**	Os08g42580.1	F: ATGCCTCCAGAGTATGCT	**127**
		R: CACTTGAAGATTCGGTTGAT	
**99**	Os07g46990.1/2	F: ACCACACTTCAATCCTACT	**265**
		R: ATGATTCCGCAAGCAACT	
**101**	Os11g09280.1/2	F: GTTGTGGTCGCTGATAACG	**121**
		R: GCTGCCTCATCCAAGATT	
**Actin**	Os11g06390.1	F: GAGTATGATGAGTCGGGTCCAG	**122**
		R: ACACCAACAATCCCAAACAGAG'	
