# Supplementary material for: Analysis of the Proteins Secreted from the Oryza meyeriana Suspension-Cultured Cells Induced by Xanthomonas oryzae pv. oryzae
Source: PLoS One. 2016 May 19;11(5):e0154793. doi: 10.1371/journal.pone.0154793 (PMC4873123; doi:10.1371/journal.pone.0154793)
Supplement: S2 Table — (PDF) [file pone.0154793.s003.pdf]

S2 Table . Primers used for QRT-PCR to detect the mRNA of peroxidases of *Oryza meyeriana*

**Spot No**	**Protein name**	**RGAP Accession number**	**Sequence (5'-3')**	**Product size (bp)**
M52	Peroxidase 4	Os07g0677100-01	ACGCCCTACTCCTTCGACAACGC	168
			AAGCCTGATCTGTCCCTGGCTCCC	
M111	Peroxidase 5	Os07g0665200-01	GACCTCTGTGACGGGAAGTGTCT	189
			CATGGCGGTTCTCATCTTGTGG	
Mock	Actin	Os11g06390.1	GAGTATGATGAGTCGGGTCCAG	125
			ACACCAACAATCCCAAACAGAG'	
